# Management of Suspected α-Pyrrolidinoisohexanophenone (α-PiHP)-Related Hyperthermia in a Young Adult: A Case Report

**DOI:** 10.7759/cureus.70708

**Published:** 2024-10-02

**Authors:** Thibaud T'kind, Pierre Baptiste Vialatte, Claire Roger, Laysa Saadi, Laurent Muller

**Affiliations:** 1 Intensive Care Unit, Centre Hospitalier Universitaire de Nîmes, Nîmes, FRA; 2 Department of Critical Care Medicine, Centre Hospitalier Universitaire de Nîmes, Nîmes, FRA; 3 Department of Critical Care Medicine, Université de Montpellier, Montpellier, FRA

**Keywords:** alpha-pihp, dantrolene, drug-induced hyperthermia, intoxication, synthetic cathinones

## Abstract

Synthetic cathinones, commonly known as "bath salts," have been increasingly implicated in severe health incidents. α-Pyrrolidinoisohexanophenone (α-PiHP) is one of the substances for which clinical data remain limited. In this article, we report a case of a 32-year-old male patient who ingested five grams of α-PiHP in a suicide attempt, resulting in hyperthermia and severe complications, including rhabdomyolysis and acute kidney injury. Despite the lack of confirmation for α-PiHP intoxication in toxicology screens, the patient's reported history was strongly suggestive. Considering a diagnostic uncertainty between serotonin syndrome and sympathomimetic toxidrome, and given the unavailability of cyproheptadine, dantrolene was administered to control the hyperthermia, resulting in a prompt and effective reduction in core body temperature. This case highlights the potential utility of dantrolene in treating hyperthermia induced by synthetic cathinones.

## Introduction

At the end of 2021, 162 synthetic cathinones were identified and considered the second most seized drugs in the group of new psychoactive substances [1,2].

They all derive from a parent compound, cathinone, which is a psychoactive substance found in Catha edulis, a plant native to East Africa and the Arabian Peninsula, where it is locally consumed for its psychoactive properties [2].

They were first synthesized in the 1920s as a potential medicinal product aimed at treating depression and suppressing appetite [3]. However, their structure was easily modified to bypass legal controls and designed to mimic the effects of more traditional psychostimulants such as methamphetamine or cocaine [2,4]. Commonly known as inexpensive 'bath salts,' they are produced in the form of crystals or powders and can be consumed by inhalation, orally, or intravenously [2,5].

Their psychoactive effects result from interacting with dopamine, serotonin, and norepinephrine transporters, exhibiting varying levels of affinity and selectivity. They all appear to be potent inhibitors of norepinephrine, but the effects on the reuptake or release of dopamine and serotonin seem to be heterogeneous, which may explain the variable clinical outcomes [6].

Consumers seek increased self-confidence, energy, psychophysical activity, enhanced sensory perception, improved concentration, and a sense of euphoria and well-being. However, these substances are also responsible for cardiovascular effects (tachycardia, increased blood pressure, chest pain, cardiac arrest), neurological effects (agitation, anxiety, hallucinations, paranoia, seizures), and other issues such as rhabdomyolysis, hyperthermia, renal toxicity, or death [2,3].

Among the numerous synthetic cathinones, there is α-pyrrolidinoisohexanophenone (α-PiHP), an analogue of alpha-pyrrolidinohexanophenone (α-PHP), first reported in 2016. Sold in powder or pill form, it acts as a psychomotor stimulant by inhibiting norepinephrine and dopamine reuptake, with potency exceeding that of cocaine, methamphetamine, or methcathinones. However, its ability to inhibit serotonin reuptake is negligible [1,7,8].

Only limited epidemiological data are available on this substance. Reports from the Drug Analysis Service in the United States indicate that it may be present in several drugs without consumers' knowledge. No clinical studies have been identified, and the available data come from analyses of discussions on forums dedicated to illicit substances. According to the information, the desired effects begin 30 to 60 minutes after oral ingestion, with durations of action ranging from 1 to 5 hours depending on the route of administration, and post-use effects lasting 6 to 12 hours [7,8].

In this clinical case report, we describe our experience with a suspected α-PiHP intoxication. The patient exhibited hyperthermia, which was treated with dantrolene. Currently, there is a lack of data on this substance, its clinical effects, and management of intoxication cases. By detailing our experience, we aim to open the discussion about the dangerous effects of this drug and how hyperthermia can be managed in such cases.

## Case presentation

A 32-year-old man was admitted to the emergency room following a suicide attempt in which he ingested five grams of α-PiHP more than double his daily dose and jumped from the first floor.

The patient’s relevant medical history includes borderline personality disorder and a long history of substance abuse, with past, non-active use of cocaine, cannabis, and amphetamines and active use of α-PiHP by inhalation for the past three years with daily consumption of approximately two grams over the last year.

On initial clinical evaluation, he exhibited bilateral otorrhagia, a right eyelid hematoma, and a Glasgow coma scale score of 15 with bilateral reactive mydriasis. Hemodynamic or respiratory parameters were stable. A full-body CT scan revealed a frontotemporal contusion, an undisplaced pelvic fracture, a displaced fracture of the left collarbone, pulmonary contusions, and a petrous bone fracture. The injury severity score was measured at nine and no surgical intervention was required.

Initial laboratory findings revealed elevated creatine phosphokinase (3573 ui/l), mildly elevated aminotransferase levels (aspartate aminotransferase (ASAT): 114 UI/L; Alanine aminotransferase (ALAT): 123 UI/L) with normal blood arterial lactate (1.4 mmol/l), urea and creatinine levels (6.4 mmol/l and 99 µmol/l, respectively) without any electrolyte disturbances. Urine toxicology screens were positive for cocaine (≥ 300 ng/mL), benzodiazepines (≥ 300 ng/ml), and amphetamines (≥ 500 ng/mL). Although α-PiHP screening is theoretically available at the institution, it is not included in the emergent standard toxicology screening and was therefore not performed in the emergency setting. We could not obtain a dosage a posteriori.

Treatment in the emergency room included the administration of crystalloids (10ml/kg) and analgesics, consisting of a single dose of acetaminophen and multiple doses of morphine.

After several hours of clinical monitoring, the patient was transferred to the intensive care unit due to the onset of agitation, confusion, tachycardia, hypertension, hypoxemia, diaphoresis, and hyperthermia reaching 41.5°c. No choreiform movement or muscular rigidity was observed.

Blood tests indicated markedly highly elevated creatine phosphokinase levels (158 000 UI/l), elevated urea and creatinine levels (31.8 mmol/l and 518 µmol/l respectively) with significantly elevated aminotransferase levels (aspartate aminotransferase (ASAT): 1332 UI/L; alanine aminotransferase (ALAT): 249 UI/L). Blood arterial lactate remained normal (0.9 mmol/l).

Symptomatic measures were implemented to manage rhabdomyolysis, KDIGO stage 3 acute kidney injury, and hyperthermia. The treatment regimen included N-acetylcysteine for hepatic protection along with crystalloid fluids and bicarbonate administration to address rhabdomyolysis

**Figure 1:**
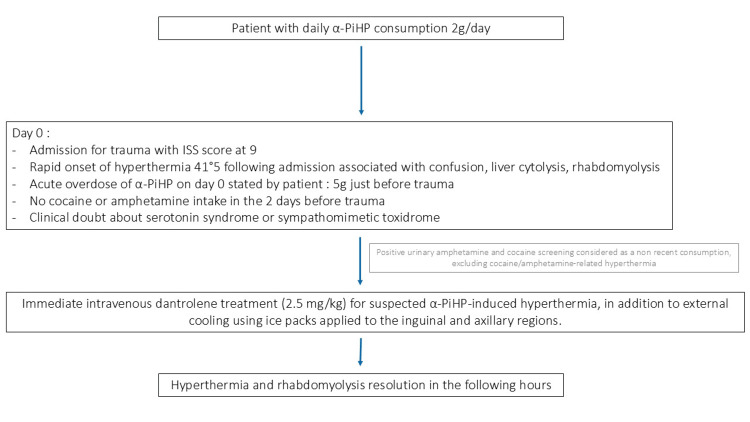
Flowchart illustrating the clinical management of a patient with suspected α-PiHP-induced hyperthermia. α-PiHP: α-Pyrrolidinoisohexanophenone

Given the uncertainty between serotonin syndrome and a sympathomimetic toxidrome, and the unavailability of cyproheptadine in our unit to manage hyperthermia potentially induced by serotonin syndrome, he received 2.5 mg/kg of dantrolene along with external cooling through the application of ice packs to the inguinal and axillary folds (Figure 1). His core temperature decreased to 37.7°C within three hours after dantrolene administration alongside the resolution of hypertension, tachycardia, and diaphoresis.

The patient remained in the intensive care unit for 15 days. His stay was marked by the acute kidney injury classified as KDIGO stage 3, with a blood peak creatinine level of 700 µmol/l, which gradually resolved without the need for continuous renal replacement therapy. Confusion and agitation persisted for several days with a follow-up brain scan at five days showing no progression of the initial lesions to account for the altered mental state. Additionally, he developed nosocomial pneumonia due to Enterobacter cloacae which was successfully treated with cefepime. The patient was discharged to another service with a follow-up plan in place for his addictive behaviors.

## Discussion

To our knowledge, the only available recommendation for treating hyperthermia induced by novel psychoactive substances, including cathinones, suggests the administration of oral cyproheptadine in combination with symptomatic treatment (cooling, curare, and orotracheal intubation) [9]. However, this recommendation is based solely on a single case report [10].

This clinical case highlights our approach to managing suspected α-PiHP-induced hyperthermia with dantrolene in the context of acute-on-chronic poisoning.

Although α-PiHP levels were not measured at any point during the hospitalization, we strongly suspected α-PiHP poisoning based on the patient's medical history, which confirmed the ingestion of five grams of α-PiHP-more than twice his usual daily dose without any other substance intake in the past 24 hours.

Dantrolene was selected for several reasons. First, the suspicion of α-PiHP intoxication. As previously mentioned, synthetic cathinones exhibit varying affinities for noradrenaline, dopamine, and serotonin transporters, depending on the specific type of cathinone [6,11]. They can therefore be responsible for hyperthermia induced by a serotonin syndrome or a sympathomimetic toxidrome [2,12]. In vitro α-PiHP seems to act as a potent inhibitor of norepinephrine and dopamine reuptake with a lower effect on the serotonin reuptake and level modulation [8,13]. In the present case, the absence of clonia or muscle rigidity does not support serotonin syndrome but mydriasis, sinus tachycardia, hypertension, confusion, and diaphoresis can be found in both serotonin syndrome and sympathomimetic toxidrome, not allowing us to distinguish between the two [14,15].

The second reason was the rapid availability of dantrolene, which is stocked in our unit for the treatment of malignant hyperthermia. Cyproheptadine, on the other hand, is difficult to obtain in French hospitals and can only be administered orally or with a nasogastric tube, which decreases the rapidity of efficacy in a life-threatening situation. Dantrolene is generally well-tolerated in single-dose administration, with rare serious side effects in the context of acute treatment of malignant hyperthermia [16].

**Figure 2:**
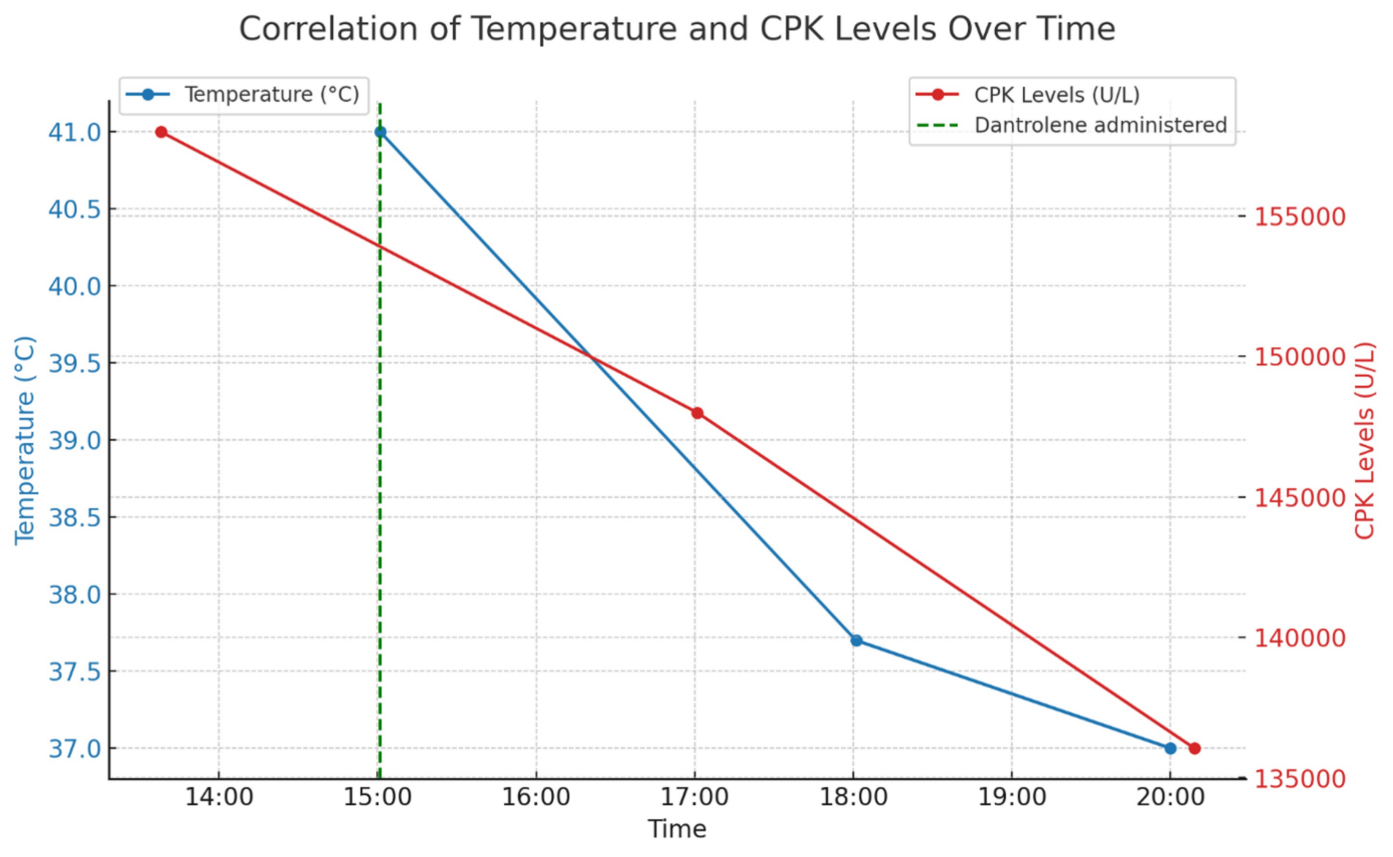
Evolution of temperature reduction and CPK levels over time following dantrolene administration This figure illustrates  the decrease in body temperature (blue line) and the reduction in creatine phosphokinase (CPK) levels (red line) over time in response to the administration of dantrolene. The administration time of dantrolene is marked by a green dashed vertical line at 15:00. The left y-axis represents the body temperature in degrees Celsius, while the right y-axis represents the CPK levels in units per liter (U/L). The observed decrease in both temperature and CPK levels following the dantrolene intervention suggests a successful therapeutic response.

Despite the clinical uncertainty between serotonin syndrome and a sympathomimetic toxidrome, dantrolene showed clinical efficacy in this suspected α-PiHP-induced hyperthermia case, resulting in a rapid and sustained decrease in both temperature and creatine phosphokinase (CPK) levels (Figure 2).

The reported mechanisms of dantrolene action include action on ryanodine receptors in skeletal muscle, blocking calcium release from the sarcoplasmic reticulum, thereby reducing muscle contraction. This results in a peripheral effect through direct muscle relaxation [16,17]. Although its use is the gold standard for treatment of anesthetic malignant hyperthermia [16], this mechanism could explain his efficiency in the management of hyperthermia induced by an adrenergic toxidrome (suspected in this clinical case) where an excessive muscular activity coupling with vasoconstriction can lead to elevated body temperature [12].

In hyperthermia induced by a serotonin syndrome, dantrolene does not appear to be effective, and many authors, based on limited evidence, suggest the use of cyproheptadine, a 5HT2A receptor antagonist, for its management [18,19]. However, in multiple cases of hyperthermia induced by 3,4-methylenedioxymethamphetamine (MDMA), which inhibits the reuptake of several neurotransmitters, particularly serotonin, dantrolene seems to be efficient as well as the cyproheptadine [10,20-22].

Like cathinones [2,4], the action of MDMA on multiple neurotransmitters [23], not just serotonin, could potentially explain why dantrolene is effective in these cases of intoxication, despite its apparent ineffectiveness in hyperthermia induced by pure serotonin syndrome [19]. However, further studies are needed to confirm this hypothesis.

Cocaine and amphetamine in the urine toxicology screening can be detected several days after their consumption. This explains the positivity for both drugs at admission [24,25]. It has been hypothesized that hyperthermia cocaine/amphetamine-related syndrome could be excluded in this specific case and α-PiHP was considered as the sole drug that could explain the observed hyperthermia because the patient clearly declared that he didn’t have cocaine or amphetamine consumption before trauma, but only α-PiHP.

This case report inherently carries the limitations of a single-case study, which preclude the establishment of causal relationships. Additionally, the absence of α-PiHP serum levels and the presence of cocaine and amphetamines in the urine limit our ability to definitively attribute the hyperthermia to α-PiHP. However, based on the patient's anamnesis at the admission and after, we strongly suspect α-PiHP intoxication. Even in the case of a mixed intoxication, this report raises important questions about the use of dantrolene in managing hyperthermia induced by illicit substances, including cathinones, and contributes valuable information on α-PiHP, a substance for which limited data currently exists.

## Conclusions

This case report suggests a potential beneficial effect of dantrolene in hyperthermia induced by acute intoxication with α-PiHP. Given the limited data on α-PiHP and other novel psychoactive substances, this case underscores the need for further research into the mechanisms of hyperthermia in drug intoxication and the optimal therapeutic interventions, particularly concerning the role of dantrolene in such scenarios.
